# Supplementary material for: Decoding the genomic landscape of chromatin-associated biomolecular condensates
Source: Nat Commun. 2024 Aug 13;15:6952. doi: 10.1038/s41467-024-51426-2 (PMC11322608; doi:10.1038/s41467-024-51426-2)
Supplement: Supplementary file 1 — Supplementary Information [file 41467_2024_51426_MOESM1_ESM.pdf]

Supplementary Figures

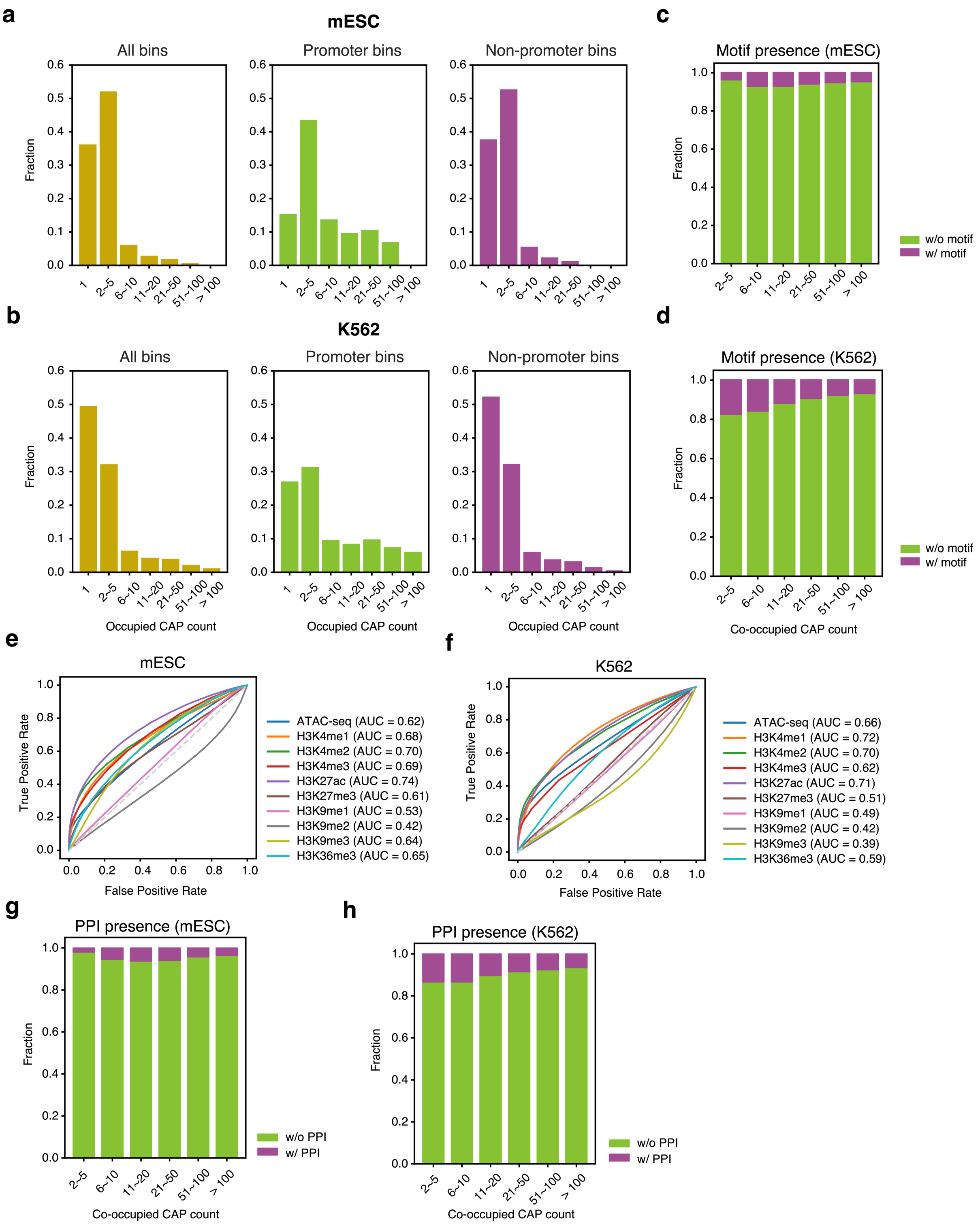

Supplementary Fig. 1. Predict chromatin-associated biomolecular condensates with CondSigDetector.

a, b. Occupancy count of CAPs at 1-kb consecutive bins (all regions, promoter regions and non-promoter regions) in mESC (**a**) and K562 (**b**). **c, d.** The stacked bar plot showing the fraction of co-occupied CAPs with or without the presence of the DNA-binding motif at the corresponding bins in mESC (**c**) and K562 (**d**). See methods for more details about motif scan. **e, f.** Receiver operator characteristic (ROC) curve analysis for the association between the presence of co-occupancy events (labeled as 1) or solo-occupancy events (labeled as 0) and epigenetic modification signals at 1-kb bins. ROC curve analysis was performed using python package scikit-learn (v1.1.3)[1]. ChIP-seq data for histone modifcations were collected from the our previous study[2], ATAC-seq data in K562 and mESC were from previous studies (GSE71218[3], GSE70482[4]), and signal tracks were downloaded from Cistrome Data Browser[5]. **g, h.** The stacked bar plot showing the fraction of co-occupied CAPs with or without support of physical protein-protein interaction (PPI). Source data are provided as a Source Data file.

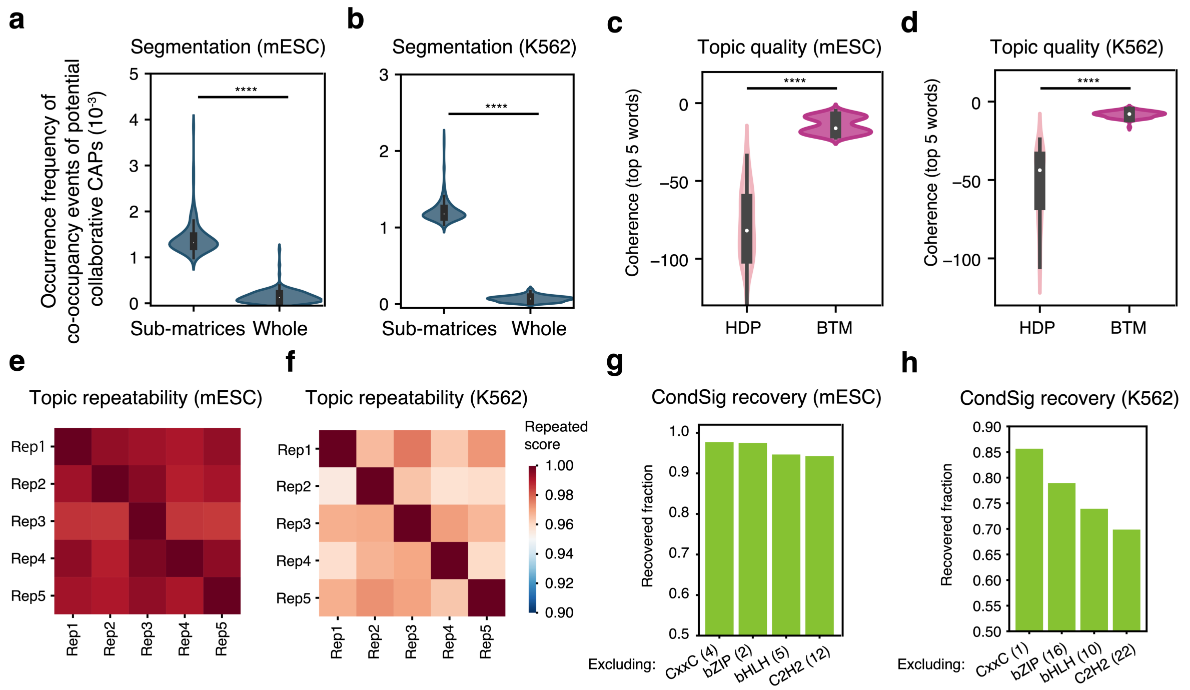

Supplementary Fig. 2. The performance of CondSigDetector.

a, b. Violin plots showing the occurrence frequency of co-occupancy events of partial highly co-occupied CAPs in sub-matrices and in the entire matrix in mESC (**a**) and K562 (**b**), demonstrating a significant increase of occurrence frequency by segmentation. See methods for more details about partial highly co-occupied CAPs. Significance between groups was evaluated by a two-sided Welch's *t*-test, **** represents p-value < 1×10^{-4}. Sample size for comparison is 159 for mESC and 215 for K562. **c, d.** Violin plots showing topic coherence to compare the quality of topics learned from HDP (Hierarchical Dirichlet Process, topic model used in RMD[6]) and BTM (biterm topic model[7], topic model used in this study). Significance between groups was evaluated by a two-sided Welch's *t*-test, **** represents p-value < 1×10^{-4}. Sample size for comparison is 101 for mESC and 93 for K562. **e, f.** Heatmaps showing repeatability of the learned topic among 5 different replicate trials in mESC (**e**) and K562 (**f**). For this analysis, the top 5 words of topics were selected to measure repeatability. Topics that shared more than 3 identical words in pairwise comparisons were considered to be repeated. The colours represent the fraction of topics in a given trial that could be replicated in another trial. **g, h.** The stacked bar plots showing fractions of original CondSigs recovered by prediction trials with excluding CAPs of the certain transcription factor family from the dataset. The name of family and the number of CAPs excluded from each family were labelled in *X*-axis. Simiar to the repeatability of topics, the top 5 CAPs of CondSigs were selected to measure recovery

level. CondSigs that shared more than 3 identical CAPs in pairwise comparisons were considered to be recovered. Source data are provided as a Source Data file.

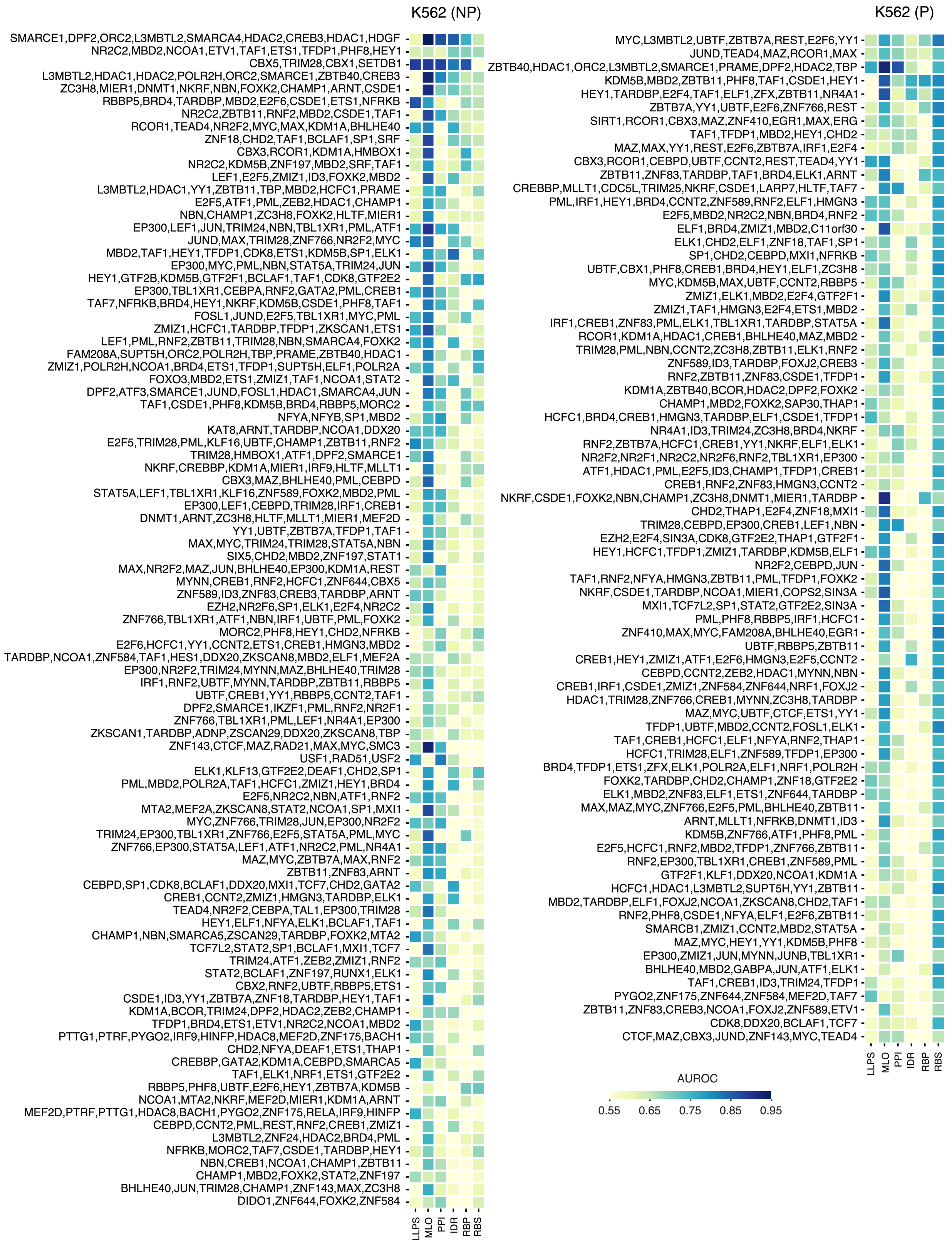
K562 (NP)
SMARCE1,DPF2,ORC2,L3MBTL2,SMARCA4,HDAC2,CREB3,HDAC1,HDGF
NR2C2,MBD2,NCOA1,ETV1,TAF1,ETS1,TFDP1,PHF8,HEY1
CBX5,TRIM28,CBX1,SETDB1
L3MBTL2,HDAC1,HDAC2,POLR2H,ORC2,SMARCE1,ZBTB40,CREB3
ZC3H8,MIER1,DNMT1,NKRF,NBN,FOXK2,CHAMP1,ARNT,CSDE1
RBBP5,BRD4,TARDBP,MBD2,E2F6,CSDE1,ETS1,NFRKB
NR2C2,ZBTB11,RNF2,MBD2,CSDE1,TAF1
RCOR1,TEAD4,NR2F2,MYC,MAX,KDM1A,BHLHE40
ZNF18,CHD2,TAF1,BCLAF1,SP1,SRF
CBX3,RCOR1,KDM1A,HMBOX1
NR2C2,KDM5B,ZNF197,MBD2,SRF,TAF1
LEF1,E2F5,ZMIZ1,ID3,FOXK2,MBD2
L3MBTL2,HDAC1,YY1,ZBTB11,TBP,MBD2,HCFC1,PRAME
E2F5,ATF1,PML,ZEB2,HDAC1,CHAMP1
NBN,CHAMP1,ZC3H8,FOXK2,HLTF,MIER1
EP300,LEF1,JUN,TRIM24,NBN,TBL1XR1,PML,ATF1
JUND,MAX,TRIM28,ZNF766,NR2F2,MYC
MBD2,TAF1,HEY1,TFDP1,CDK8,ETS1,KDM5B,SP1,ELK1
EP300,MYC,PML,NBN,STAT5A,TRIM24,JUN
HEY1,GTF2B,KDM5B,GTF2F1,BCLAF1,TAF1,CDK8,GTF2E2
EP300,TBL1XR1,CEBPA,RNF2,GATA2,PML,CREB1
TAF7,NFRKB,BRD4,HEY1,NKRF,KDM5B,CSDE1,PHF8,TAF1
FOSL1,JUND,E2F5,TBL1XR1,MYC,PML
ZMIZ1,HCFC1,TARDBP,TFDP1,ZKSCAN1,ETS1
LEF1,PML,RNF2,ZBTB11,TRIM28,NBN,SMARCA4,FOXK2
FAM208A,SUPT5H,ORC2,POLR2H,TBP,PRAME,ZBTB40,HDAC1
ZMIZ1,POLR2H,NCOA1,BRD4,ETS1,TFDP1,SUPT5H,ELF1,POLR2A
FOXO3,MBD2,ETS1,ZMIZ1,TAF1,NCOA1,STAT2
DPF2,ATF3,SMARCE1,JUND,FOSL1,HDAC1,SMARCA4,JUN
TAF1,CSDE1,PHF8,KDM5B,BRD4,RBBP5,MORC2
NFYA,NFYB,SP1,MBD2
KAT8,ARNT,TARDBP,NCOA1,DDX20
E2F5,TRIM28,PML,KLF16,UBTF,CHAMP1,ZBTB11,RNF2
TRIM28,HMBOX1,ATF1,DPF2,SMARCE1
NKRF,CREBBP,KDM1A,MIER1,IRF9,HLTF,MLLT1
CBX3,MAZ,BHLHE40,PML,CEBPD
STAT5A,LEF1,TBL1XR1,KLF16,ZNF589,FOXK2,MBD2,PML
EP300,LEF1,CEBPD,TRIM28,IRF1,CREB1
DNMT1,ARNT,ZC3H8,HLTF,MLLT1,MIER1,MEF2D
YY1,UBTF,ZBTB7A,TFDP1,TAF1
MAX,MYC,TRIM24,TRIM28,STAT5A,NBN
SIX5,CHD2,MBD2,ZNF197,STAT1
MAX,NR2F2,MAZ,JUN,BHLHE40,EP300,KDM1A,REST
MYNN,CREB1,RNF2,HCFC1,ZNF644,CBX5
ZNF589,ID3,ZNF83,CREB3,TARDBP,ARNT
EZH2,NR2F6,SP1,ELK1,E2F4,NR2C2
ZNF766,TBL1XR1,ATF1,NBN,IRF1,UBTF,PML,FOXK2
MORC2,PHF8,HEY1,CHD2,NFRKB
E2F6,HCFC1,YY1,CCNT2,ETS1,CREB1,HMGN3,MBD2
TARDBP,NCOA1,ZNF584,TAF1,HES1,DDX20,ZKSCAN8,MBD2,ELF1,MEF2A
EP300,NR2F2,TRIM24,MYNN,MAZ,BHLHE40,TRIM28
IRF1,RNF2,UBTF,MYNN,TARDBP,ZBTB11,RBBP5
UBTF,CREB1,YY1,RBBP5,CCNT2,TAF1
DPF2,SMARCE1,IKZF1,PML,RNF2,NR2F1
ZNF766,TBL1XR1,PML,LEF1,NR4A1,EP300
ZKSCAN1,TARDBP,ADNP,ZSCAN29,DDX20,ZKSCAN8,TBP
ZNF143,CTCF,MAZ,RAD21,MAX,MYC,SMC3
USF1,RAD51,USF2
ELK1,KLF13,GTF2E2,DEAF1,CHD2,SP1
PML,MBD2,POLR2A,TAF1,HCFC1,ZMIZ1,HEY1,BRD4
E2F5,NR2C2,NBN,ATF1,RNF2
MTA2,MEF2A,ZKSCAN8,STAT2,NCOA1,SP1,MXI1
MYC,ZNF766,TRIM28,JUN,EP300,NR2F2
TRIM24,EP300,TBL1XR1,ZNF766,E2F5,STAT5A,PML,MYC
ZNF766,EP300,STAT5A,LEF1,ATF1,NR2C2,PML,NR4A1
MAZ,MYC,ZBTB7A,MAX,RNF2
ZBTB11,ZNF83,ARNT
CEBPD,SP1,CDK8,BCLAF1,DDX20,MXI1,TCF7,CHD2,GATA2
CREB1,CCNT2,ZMIZ1,HMGN3,TARDBP,ELK1
TEAD4,NR2F2,CEBPA,TAL1,EP300,TRIM28
HEY1,ELF1,NFYA,ELK1,BCLAF1,TAF1
CHAMP1,NBN,SMARCA5,ZSCAN29,TARDBP,FOXK2,MTA2
TCF7L2,STAT2,SP1,BCLAF1,MXI1,TCF7
TRIM24,ATF1,ZEB2,ZMIZ1,RNF2
STAT2,BCLAF1,ZNF197,RUNX1,ELK1
CBX2,RNF2,UBTF,RBBP5,ETS1
CSDE1,ID3,YY1,ZBTB7A,ZNF18,TARDBP,HEY1,TAF1
KDM1A,BCOR,TRIM24,DPF2,HDAC2,ZEB2,CHAMP1
TFDP1,BRD4,ETS1,ETV1,NR2C2,NCOA1,MBD2
PTTG1,PTRF,PYGO2,IRF9,HINFP,HDAC8,MEF2D,ZNF175,BACH1
CHD2,NFYA,DEAF1,ETS1,THAP1
CREBBP,GATA2,KDM1A,CEBPD,SMARCA5
TAF1,ELK1,NRF1,ETS1,GTF2E2
RBBP5,PHF8,UBTF,E2F6,HEY1,ZBTB7A,KDM5B
NCOA1,MTA2,NKRF,MEF2D,MIER1,KDM1A,ARNT
MEF2D,PTRF,PTTG1,HDAC8,BACH1,PYGO2,ZNF175,RELA,IRF9,HINFP
CEBPD,CCNT2,PML,REST,RNF2,CREB1,ZMIZ1
L3MBTL2,ZNF24,HDAC2,BRD4,PML
NFRKB,MORC2,TAF7,CSDE1,TARDBP,HEY1
NBN,CREB1,NCOA1,CHAMP1,ZBTB11
CHAMP1,MBD2,FOXK2,STAT2,ZNF197
BHLHE40,JUN,TRIM28,CHAMP1,ZNF143,MAX,ZC3H8
DIDO1,ZNF644,FOXK2,ZNF584
LLPS
MLO
PPI
IDR
RBP
RBS
K562 (P)
MYC,L3MBTL2,UBTF,ZBTB7A,REST,E2F6,YY1
JUND,TEAD4,MAZ,RCOR1,MAX
ZBTB40,HDAC1,ORC2,L3MBTL2,SMARCE1,PRAME,DPF2,HDAC2,TBP
KDM5B,MBD2,ZBTB11,PHF8,TAF1,CSDE1,HEY1
HEY1,TARDBP,E2F4,TAF1,ELF1,ZFX,ZBTB11,NR4A1
ZBTB7A,YY1,UBTF,E2F6,ZNF766,REST
SIRT1,RCOR1,CBX3,MAZ,ZNF410,EGR1,MAX,ERG
TAF1,TFDP1,MBD2,HEY1,CHD2
MAZ,MAX,YY1,REST,E2F6,ZBTB7A,IRF1,E2F4
CBX3,RCOR1,CEBPD,UBTF,CCNT2,REST,TEAD4,YY1
ZBTB11,ZNF83,TARDBP,TAF1,BRD4,ELK1,ARNT
CREBBP,MLLT1,CDC5L,TRIM25,NKRF,CSDE1,LARP7,HLTF,TAF7
PML,IRF1,HEY1,BRD4,CCNT2,ZNF589,RNF2,ELF1,HMGN3
E2F5,MBD2,NR2C2,NBN,BRD4,RNF2
ELF1,BRD4,ZMIZ1,MBD2,C11orf30
ELK1,CHD2,ELF1,ZNF18,TAF1,SP1
SP1,CHD2,CEBPD,MXI1,NFRKB
UBTF,CBX1,PHF8,CREB1,BRD4,HEY1,ELF1,ZC3H8
MYC,KDM5B,MAX,UBTF,CCNT2,RBBP5
ZMIZ1,ELK1,MBD2,E2F4,GTF2F1
ZMIZ1,TAF1,HMGN3,E2F4,ETS1,MBD2
IRF1,CREB1,ZNF83,PML,ELK1,TBL1XR1,TARDBP,STAT5A
RCOR1,KDM1A,HDAC1,CREB1,BHLHE40,MAZ,MBD2
TRIM28,PML,NBN,CCNT2,ZC3H8,ZBTB11,ELK1,RNF2
ZNF589,ID3,TARDBP,FOXJ2,CREB3
RNF2,ZBTB11,ZNF83,CSDE1,TFDP1
KDM1A,ZBTB40,BCOR,HDAC2,DPF2,FOXK2
CHAMP1,MBD2,FOXK2,SAP30,THAP1
HCFC1,BRD4,CREB1,HMGN3,TARDBP,ELF1,CSDE1,TFDP1
NR4A1,ID3,TRIM24,ZC3H8,BRD4,NKRF
RNF2,ZBTB7A,HCFC1,CREB1,YY1,NKRF,ELF1,ELK1
NR2F2,NR2F1,NR2C2,NR2F6,RNF2,TBL1XR1,EP300
ATF1,HDAC1,PML,E2F5,ID3,CHAMP1,TFDP1,CREB1
CREB1,RNF2,ZNF83,HMGN3,CCNT2
NKRF,CSDE1,FOXK2,NBN,CHAMP1,ZC3H8,DNMT1,MIER1,TARDBP
CHD2,THAP1,E2F4,ZNF18,MXI1
TRIM28,CEBPD,EP300,CREB1,LEF1,NBN
EZH2,E2F4,SIN3A,CDK8,GTF2E2,THAP1,GTF2F1
HEY1,HCFC1,TFDP1,ZMIZ1,TARDBP,KDM5B,ELF1
NR2F2,CEBPD,JUN
TAF1,RNF2,NFYA,HMGN3,ZBTB11,PML,TFDP1,FOXK2
NKRF,CSDE1,TARDBP,NCOA1,MIER1,COPS2,SIN3A
MXI1,TCF7L2,SP1,STAT2,GTF2E2,SIN3A
PML,PHF8,RBBP5,IRF1,HCFC1
ZNF410,MAX,MYC,FAM208A,BHLHE40,EGR1
UBTF,RBBP5,ZBTB11
CREB1,HEY1,ZMIZ1,ATF1,E2F6,HMGN3,E2F5,CCNT2
CEBPD,CCNT2,ZEB2,HDAC1,MYNN,NBN
CREB1,IRF1,CSDE1,ZMIZ1,ZNF584,ZNF644,NRF1,FOXJ2
HDAC1,TRIM28,ZNF766,CREB1,MYNN,ZC3H8,TARDBP
MAZ,MYC,UBTF,CTCF,ETS1,YY1
TFDP1,UBTF,MBD2,CCNT2,FOSL1,ELK1
TAF1,CREB1,HCFC1,ELF1,NFYA,RNF2,THAP1
HCFC1,TRIM28,ELF1,ZNF589,TFDP1,EP300
BRD4,TFDP1,ETS1,ZFX,ELK1,POLR2A,ELF1,NRF1,POLR2H
FOXK2,TARDBP,CHD2,CHAMP1,ZNF18,GTF2E2
ELK1,MBD2,ZNF83,ELF1,ETS1,ZNF644,TARDBP
MAX,MAZ,MYC,ZNF766,E2F5,PML,BHLHE40,ZBTB11
ARNT,MLLT1,NFRKB,DNMT1,ID3
KDM5B,ZNF766,ATF1,PHF8,PML
E2F5,HCFC1,RNF2,MBD2,TFDP1,ZNF766,ZBTB11
RNF2,EP300,TBL1XR1,CREB1,ZNF589,PML
GTF2F1,KLF1,DDX20,NCOA1,KDM1A
HCFC1,HDAC1,L3MBTL2,SUPT5H,YY1,ZBTB11
MBD2,TARDBP,ELF1,FOXJ2,NCOA1,ZKSCAN8,CHD2,TAF1
RNF2,PHF8,CSDE1,NFYA,ELF1,E2F6,ZBTB11
SMARCB1,ZMIZ1,CCNT2,MBD2,STAT5A
MAZ,MYC,HEY1,YY1,KDM5B,PHF8
EP300,ZMIZ1,JUN,MYNN,JUNB,TBL1XR1
BHLHE40,MBD2,GABPA,JUN,ATF1,ELK1
TAF1,CREB1,ID3,TRIM24,TFDP1
PYGO2,ZNF175,ZNF644,ZNF584,MEF2D,TAF7
ZBTB11,ZNF83,CREB3,NCOA1,FOXJ2,ZNF589,ETV1
CDK8,DDX20,BCLAF1,TCF7
CTCF,MAZ,CBX3,JUND,ZNF143,MYC,TEAD4
LLPS
MLO
PPI
IDR
RBP
RBS
AUROC
0.55
0.65
0.75
0.85
0.95

Supplementary Fig. 3. CondSigs in K562.

Heatmaps showing identified CondSigs in K562 at non-promoter (NP) and promoter (P) regions. Each row represents a CondSig and the row name represents the component CAPs of the given CondSig**.** Each column represents a condensation-related feature and the colours represent AUROC. Source data are provided as a Source Data file.

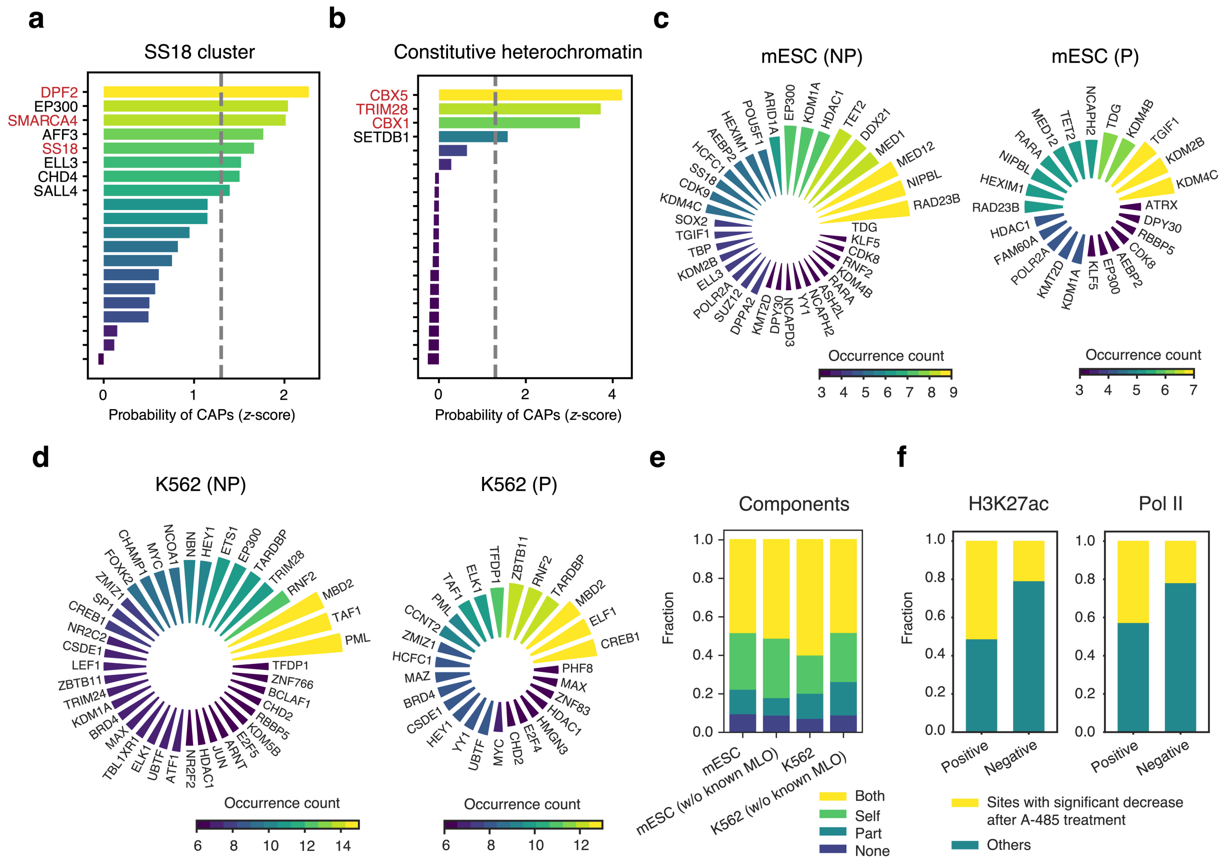

Supplementary Fig. 4. Display of CondSigs in mESC and K562.

a, b. Representative CondSigs related to known chromatin-associated biomolecular condensate, SS18 cluster (**a**), and constitutive heterochromatin (**b**). *X*-axis represent *z*-score normalized occurrence probability of component CAPs in CondSig detected by biterm topic model. Grey dashed line represents threshold determining components. Component CAPs of the CondSig were labeled and component CAPs in known chromatin-associated biomolecular condensates were highlighted in red. **c, d.** Circular bar plots showing the occurrence count of component CAPs in CondSigs in mESC (**c**) and K562 (**d**). **e.** The stacked bar plots showing the fraction of component CAPs belonging to four clusters ("Both": both PS-Self and PS-Part, "Self": Self-only, "Part": Part-only, and "None"). The fractions of component CAPs with or without known MLO memberships were shown respectively. **f.** The stacked bar plots showing the fractions of CondSig-positive / negative EP300 peaks with significant decrease in H3K27ac (left) and Pol II (right) signals after A-485 treatment. The significant decrease was defined as $\log_2$-transformed fold change (A-485 treatment versus wild type) < -1. Source data are provided as a Source Data file.

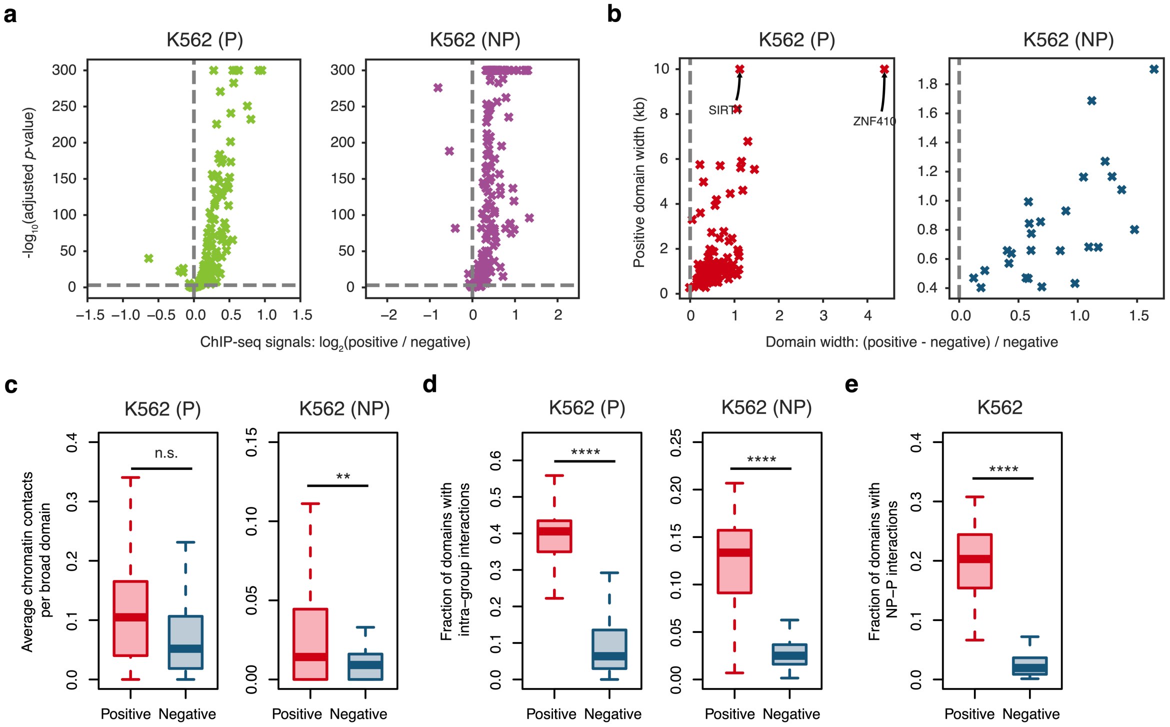

Supplementary Fig. 5. Chromatin properties of identified CondSigs.

a. Volcano plots showing concentration levels of component CAPs in CondSigs in K562. *X*-axis represents the $\log_2$-transformed fold change of ChIP-seq signals at CondSig-positive peaks compared to CondSig-negative peaks, while *Y*-axis represents the negative $\log_{10}$-transformed adjusted *p*-value. Significance between groups was evaluated by a two-sided Welch's *t*-test, and the Benjamini-Hochberg (BH) procedure was applied to adjust *p*-values for multiple testing. The vertical dashed line corresponds to fold change = 1 and the horizontal dashed line corresponds to adjusted *p*-value = 0.001. **b.** Scatter plots showing width comparison of CondSig-positive and -negative domains. *X*-axis represents the ratio to which CondSig-positive domain width exceeds CondSig-negative domain width, and *Y*-axis represents the positive domain width. Component CAPs having CondSig-positive domains exceeding 10 kb on average were labeled. **c.** Box plots showing intra-domain chromatin contacts of CondSig-positive or -negative broad domains in K562. For each component CAP, an average valid paired-end tags count in each broad domain (> 5 kb) was calculated to represent intra-domain contacts. Pol II ChIA-PET data used in the analysis was from the previous study[8]. The centre lines mark the median, the box limits indicate the 25th and 75th percentiles, and the whiskers extend to 1.5 × the interquartile range from the 25th and 75th percentiles. Significance between groups was evaluated by a one-sided Welch's *t*-test, n.s. represents non-significant, ** represents *p*-value < 0.01. Sample size for comparison is 127 for promoter regions and 26 for non-

promoter regions. **d.** Box plots showing intra-group chromatin contacts between CondSig-positive or -negative domains in K562. For each component CAP, the fraction of domains having at least one valid paired-end tag with other intra-group domains was calculated. The centre lines mark the median, the box limits indicate the 25th and 75th percentiles, and the whiskers extend to 1.5 × the interquartile range from the 25th and 75th percentiles. Significance between groups was evaluated by a one-sided Welch's *t*-test, **** represents p-value $< 1 \times 10^{-4}$. Sample size for comparison is 131 for promoter regions and 26 for non-promoter regions. **e.** Box plots showing NP (non-promoter)-P (promoter) chromatin contacts between CondSig-positive or -negative domains in K562. For each component CAP, the fraction of non-promoter domains having at least one valid paired-end tag with its promoter domains was calculated. The centre lines mark the median, the box limits indicate the 25th and 75th percentiles, and the whiskers extend to 1.5 × the interquartile range from the 25th and 75th percentiles. Significance between groups was evaluated by a one-sided Welch's *t*-test, n.s represents non-significant, * represents p-value < 0.05 and **** represents p-value $< 1 \times 10^{-4}$. Sample size for comparison is 19. Source data are provided as a Source Data file.

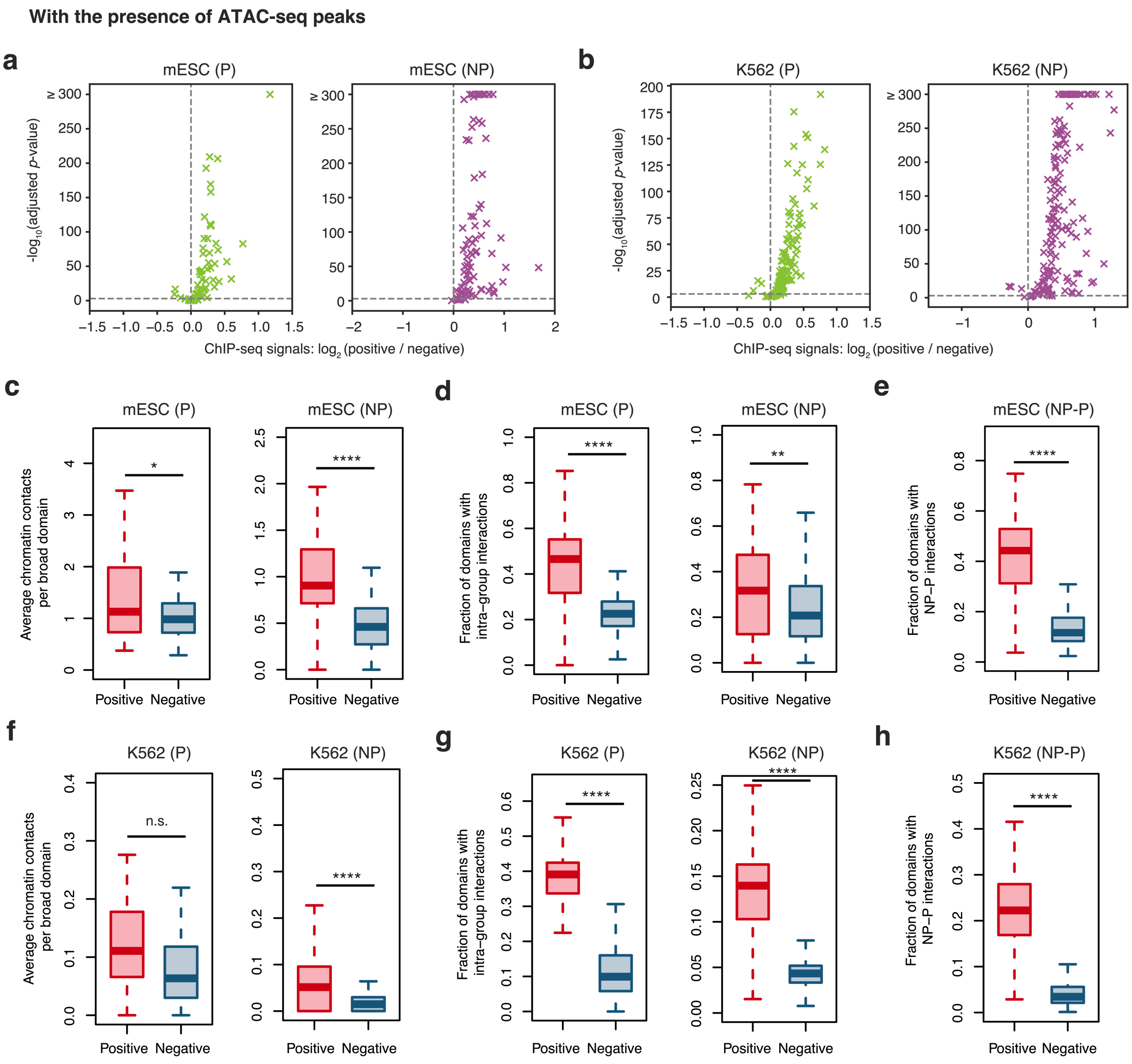

Supplementary Fig. 6. Chromatin properties of identified CondSigs (comparison at CondSig-positive / negative peaks with the presence of ATAC-seq peaks).

a, b. Volcano plots showing concentration levels of component CAPs in CondSigs in mESC (**a**) and K562 (**b**). *X*-axis represents the $\log_2$-transformed fold change of ChIP-seq signals at CondSig-positive peaks compared to CondSig-negative peaks, while *Y*-axis represents the negative $\log_{10}$-transformed adjusted *p*-value. Significance between groups was evaluated by a two-sided Welch's *t*-test, and the Benjamini-Hochberg (BH) procedure was applied to adjust *p*-values for multiple testing. The vertical dashed line corresponds to fold change = 1 and the horizontal dashed line corresponds to adjusted *p*-value = 0.001. **c.** Intra-domain chromatin contacts of CondSig-positive or -negative broad domains in mESC. For each component CAP, an average valid paired-end tags count in each broad domain (> 5 kb) was calculated to represent intra-domain contacts. The centre lines mark

the median, the box limits indicate the 25th and 75th percentiles, and the whiskers extend to 1.5 × the interquartile range from the 25th and 75th percentiles. Significance between groups was evaluated by a one-sided Welch's *t*-test, * represents *p*-value < 0.05 and **** represents p-value < 1×10^{-4}. Sample size for comparison is 61 for promoter regions and 90 for non-promoter regions. **d.** Box plots showing intra-group chromatin contacts between CondSig-positive or -negative domains in mESC. For each component CAP, the fraction of domains having at least one valid paired-end tag with other intra-group domains was calculated. The centre lines mark the median, the box limits indicate the 25th and 75th percentiles, and the whiskers extend to 1.5 × the interquartile range from the 25th and 75th percentiles. Significance between groups was evaluated by a one-sided Welch's *t*-test, ** represents *p*-value < 0.01 and **** represents p-value < 1×10^{-4}. Sample size for comparison is 63 for promoter regions and 93 for non-promoter regions. **e.** Box plots showing NP (non-promoter)-P (promoter) chromatin contacts between CondSig-positive or -negative domains in mESC. For each component CAP, the fraction of non-promoter domains having at least one valid paired-end tag with its promoter domains was calculated. The centre lines mark the median, the box limits indicate the 25th and 75th percentiles, and the whiskers extend to 1.5 × the interquartile range from the 25th and 75th percentiles. Significance between groups was evaluated by a one-sided Welch's *t*-test, n.s. represents non-significant, * represents *p*-value < 0.05, ** represents *p*-value < 0.01, *** represents p-value < 0.001 and **** represents p-value < 1×10^{-4}. Sample size for comparion is 53. **f.** Same as (**c**), but showing intra-domain chromatin contacts of CondSig-positive or -negative broad domains in K562. **g.** Same as (**d**), but showing intra-group chromatin contacts between CondSig-positive or -negative domains in K562. **h.** Same as (**e**), but showing NP (non-promoter)-P (promoter) chromatin contacts between CondSig-positive or -negative domains in K562. The samples used to derive statistics are provided in Source data. Source data are provided as a Source Data file.

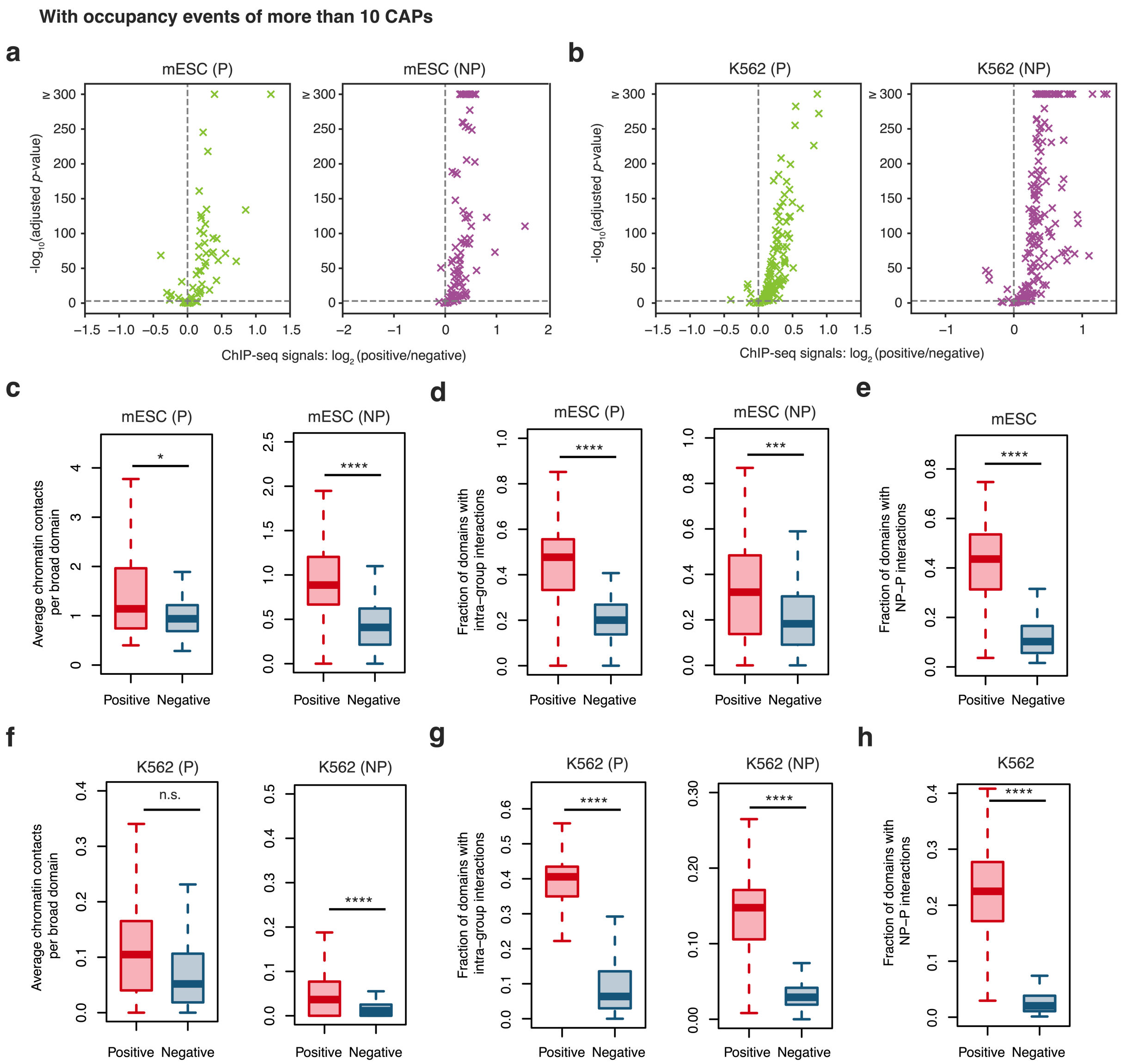

Supplementary Fig. 7. Chromatin properties of identified CondSigs (comparison at CondSig-positive / negative peaks with occupancy events of more than 10 CAPs). **a, b.** Volcano plots showing concentration levels of component CAPs in CondSigs in mESC (**a**) and K562 (**b**). *X*-axis represents the $\log_2$-transformed fold change of ChIP-seq signals at CondSig-positive peaks compared to CondSig-negative peaks, while *Y*-axis represents the negative $\log_{10}$-transformed adjusted *p*-value. Significance between groups was evaluated by a two-sided Welch's *t*-test, and the Benjamini-Hochberg (BH) procedure was applied to adjust *p*-values for multiple testing. The vertical dashed line corresponds to fold change = 1 and the horizontal dashed line corresponds to adjusted *p*-value = 0.001. **c.** Intra-domain chromatin contacts of CondSig-positive or -negative broad domains in mESC. For each component CAP, an average valid paired-end tags count in each broad

domain (> 5 kb) was calculated to represent intra-domain contacts. The centre lines mark the median, the box limits indicate the 25th and 75th percentiles, and the whiskers extend to 1.5 × the interquartile range from the 25th and 75th percentiles. Significance between groups was evaluated by a one-sided Welch's *t*-test, * represents *p*-value < 0.05 and **** represents p-value < 1×10^{-4}. Sample size for comparison is 61 for promoter regions and 90 for non-promoter regions. **d.** Box plots showing intra-group chromatin contacts between CondSig-positive or -negative domains in mESC. For each component CAP, the fraction of domains having at least one valid paired-end tag with other intra-group domains was calculated. The centre lines mark the median, the box limits indicate the 25th and 75th percentiles, and the whiskers extend to 1.5 × the interquartile range from the 25th and 75th percentiles. Significance between groups was evaluated by a one-sided Welch's *t*-test, *** represents *p*-value < 0.001 and **** represents p-value < 1×10^{-4}. Sample size for comparison is 63 for promoter regions and 93 for non-promoter regions. **e.** Box plots showing NP (non-promoter)-P (promoter) chromatin contacts between CondSig-positive or -negative domains in mESC. For each component CAP, the fraction of non-promoter domains having at least one valid paired-end tag with its promoter domains was calculated. The centre lines mark the median, the box limits indicate the 25th and 75th percentiles, and the whiskers extend to 1.5 × the interquartile range from the 25th and 75th percentiles. Significance between groups was evaluated by a one-sided Welch's *t*-test, n.s. represents non-significant, * represents *p*-value < 0.05, ** represents *p*-value < 0.01, *** represents p-value < 0.001 and **** represents p-value < 1×10^{-4}. Sample size for comparion is 53. **f.** Same as (**c**), but showing intra-domain chromatin contacts of CondSig-positive or -negative broad domains in K562. **g.** Same as (**d**), but showing intra-group chromatin contacts between CondSig-positive or -negative domains in K562. **h.** Same as (**e**), but showing NP (non-promoter)-P (promoter) chromatin contacts between CondSig-positive or -negative domains in K562. The samples used to derive statistics are provided in Source data. Source data are provided as a Source Data file.

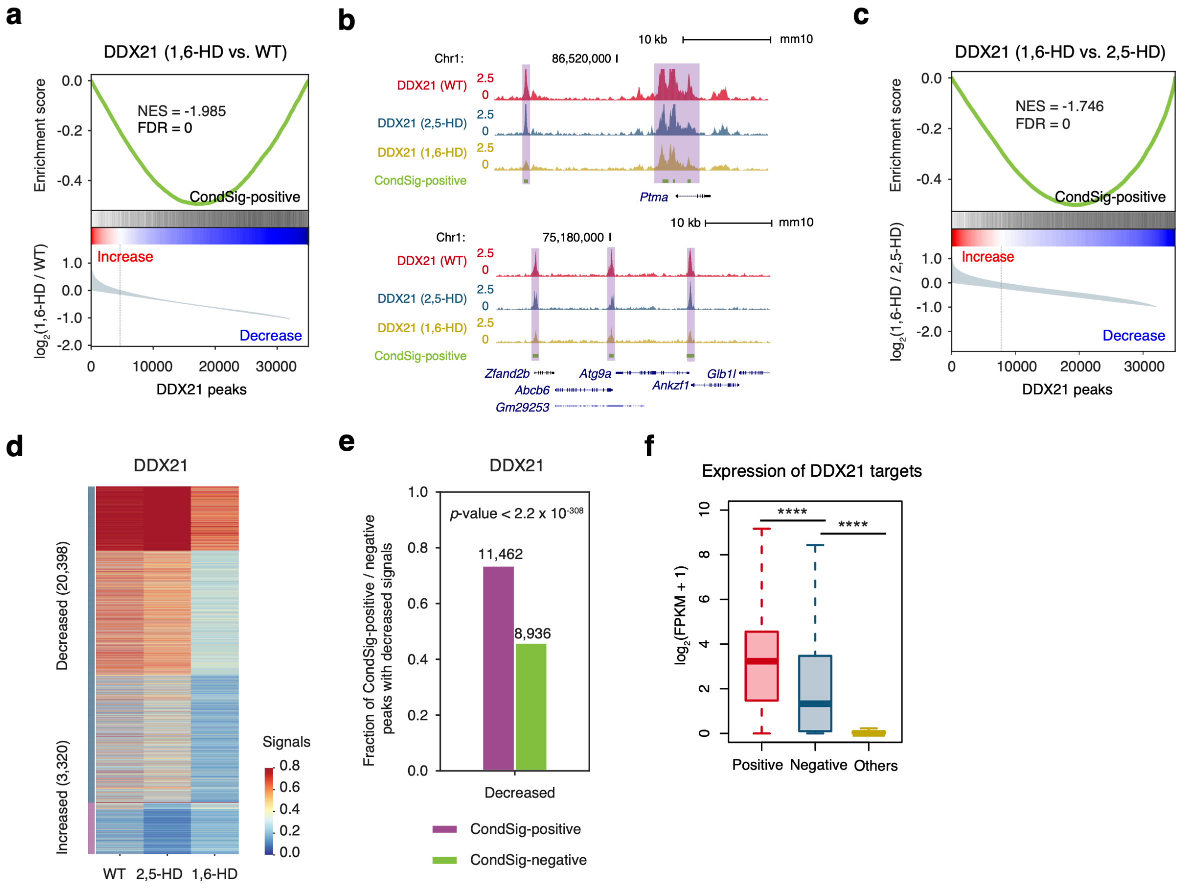

Supplementary Fig. 8. Experimental validaiton of DDX21.

a. GSEA-like analyses for CondSig-positive peaks of DDX21, with all sites (CondSig-positive and -negative peaks of DDX21) were ranked by the log_2-transformed fold change in CUT&RUN signals and annotated against the set of CondSig-positive sites. Fold changes were from 1,6-HD treatment versus wild type, and pseudo count used in fold change calculation was set to 0.1. **b.** UCSC genome browser view of representative CondSig-positive sites. Signals represent RPM and the related loci were shaded in purple. **c.** Same as (**a**), but the fold changes were from 1,6-HD treatment versus 2,5-HD treatment. **d**. Heatmap showing CUT&RUN signals for DDX21 across its peaks with changed signals (including decreased and increased peaks) when comparing 1,6-HD treatment to 2,5-HD treatment. Peaks with decreased signals (log_2-transformed fold change < -0.25) were marked in dark blue and peaks with increased signals (log_2-transformed fold change > 0.25) were marked in purple. Each group of peak was ranked based on the result of a *k*-means clustering without predetermined cluster centroids, which utilized signals from both 2,5-HD treatment and 1,6-HD treatment. **e.** Bar plots showing the fractions of CondSig-postiive / negative peaks with decreased signals out of all CondSig-positive / negative

peaks, the exact number of peaks was labelled on the top of bars. A Fisher's exact test was performed to test the significance of enrichment of CondSig-positive peaks in the group with decreased signals and *p*-value was explicitly labelled. **f.** Box plots showing expression levels of target genes of CondSig-positive DDX21 peaks, CondSig-negative DDX21 peaks and other genes. The centre lines mark the median, the box limits indicate the 25th and 75th percentiles, and the whiskers extend to 1.5 × the interquartile range from the 25th and 75th percentiles. Significance between groups was evaluated by a two-sided Welch's *t*-test, **** represents *p*-value $< 1 \times 10^{-4}$. Sample size for comparison is 11,207 for CondSig-positive target genes, 8,003 for CondSig-negative target genes and 9,437 for other genes. Source data are provided as a Source Data file.

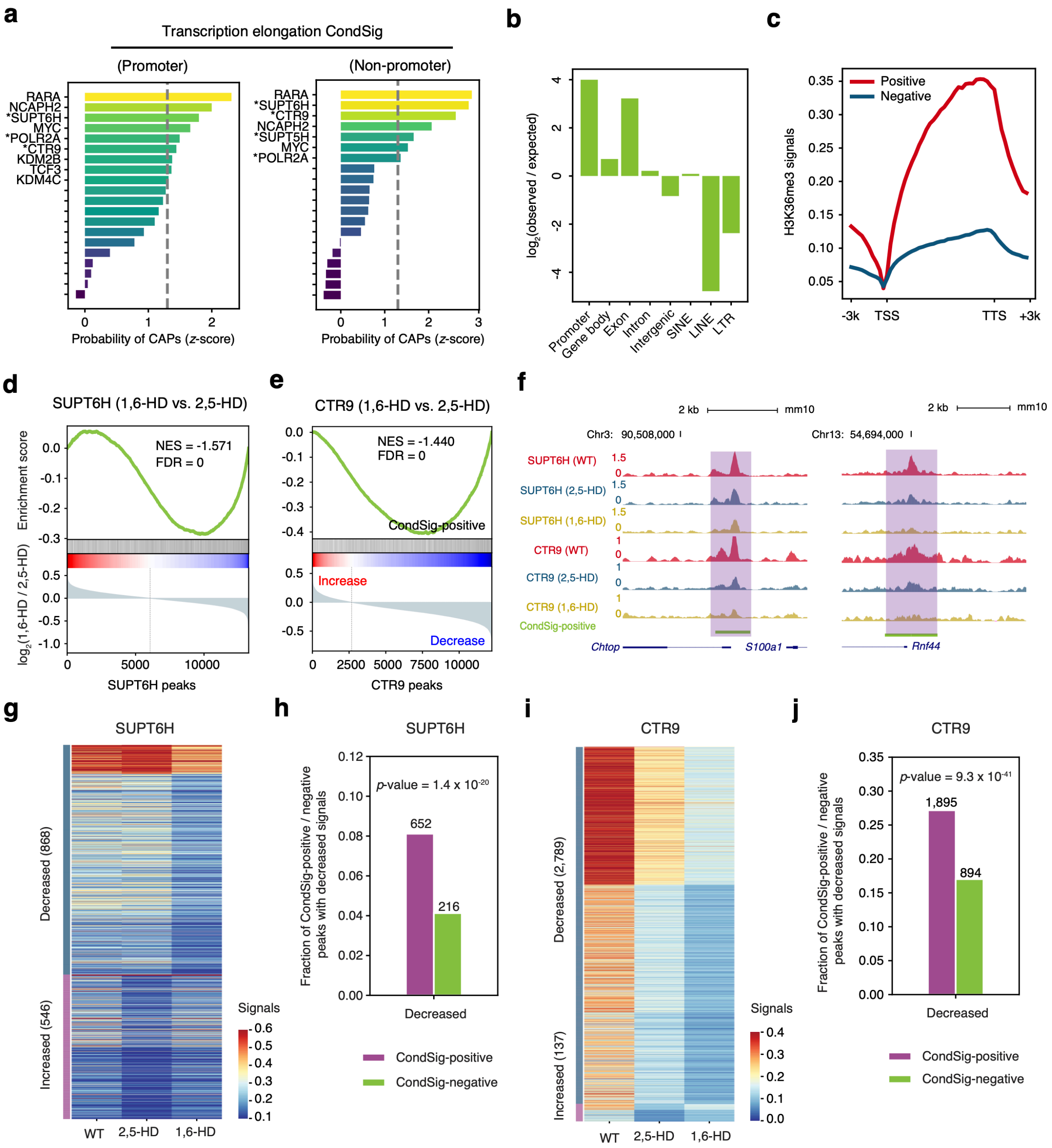

Supplementary Fig. 9. Experimental validaiton of identified CondSigs

a. Heatmap showing components of the two CondSigs. *X*-axis represents *z*-score normalized probability of CAPs in CondSig detected by biterm topic model. Grey dashed line indicates threshold determining components. SUPT6H, SUPT5H, CTR9 and POLR2A were specially labelled with *. **b.** Genomic enrichment analysis of positive sites of the two CondSigs. *Y*-axis represents the log_2-transformed observed / expected ratio. **c.** Line charts showing H3K36me3 signals at gene bodies of genes overlapping with

CondSig-positive or -negative sites. **d, e.** GSEA-like analyses for SUPT6H (**d**) and CTR9 (**e**), with all sites (CondSig-positive and -negative peaks of the CAP) were ranked by the $\log_2$-transformed fold change in CUT&RUN signals and annotated against the set of CondSig-positive peaks. Fold changes were from 1,6-HD versus 2,5-HD, and pseudo used in fold change calculations was set to 0.1. **f.** UCSC genome browser view of representative CondSig-positive sites. Signals represent RPM and the related loci were shaded in purple. **g.** Heatmap showing CUT&RUN signals for SUPT6H across its peaks with changed signals (including decreased and increased peaks) when comparing 1,6-HD treatment to 2,5-HD treatment. Peaks with decreased signals ($\log_2$-transformed fold change < -0.25) were marked in dark blue and peaks with increased signals ($\log_2$-transformed fold change > 0.25) were marked in purple. Each group of peak was ranked based on the result of a *k*-means clustering without predetermined cluster centroids, which utilized signals from both 2,5-HD treatment and 1,6-HD treatment. **h.** Bar plots showing the fractions of CondSig-postiive / negative peaks with decreased signals out of all CondSig-positive / negative peaks, the exact number of peaks was labelled on the top of bars. A Fisher's exact test was performed to test the significance of enrichment of CondSig-positive peaks in the group with decreased signals and *p*-value was explicitly labelled. **i.** Same as (**g**) but for CTR9. **j.** Same as (**h**) but for CTR9. Source data are provided as a Source Data file.

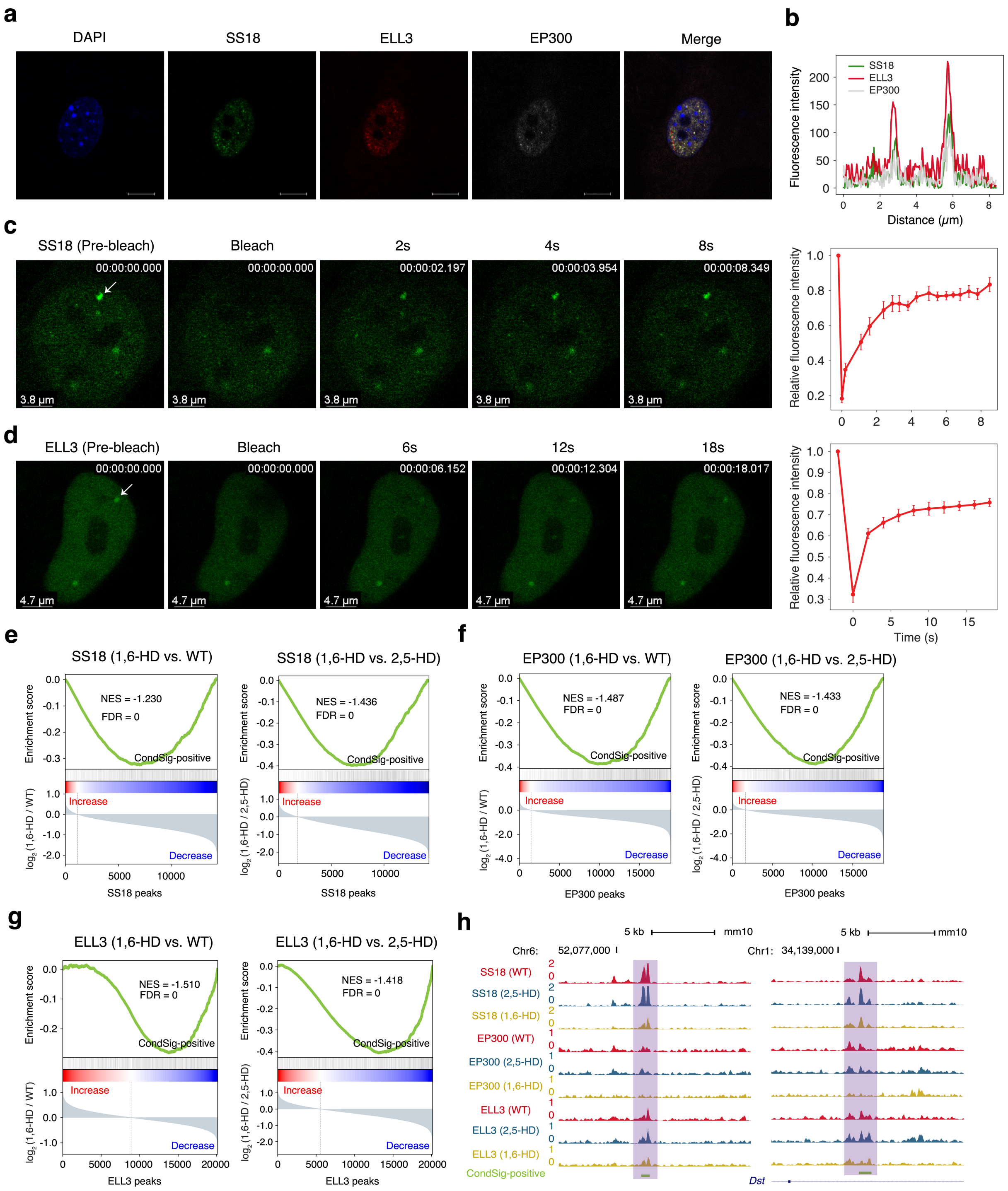

Supplementary Fig. 10. Additional experimental validaitons of identified CondSigs. **a.** Immunofluorescence images of mESC showing that SS18 (green) colocalizes with ELL3 (red) and EP300 (grey) in puncta. DNA was stained with DAPI (blue). This experimental result was consistent across two independent cell-seeding, fixation, and co-IF staining experiments (each contains three slides). Scale bar: 10 μm. Contrast ratio

adjustment was applied for the entire image of each individual colour channel. **b.** Line scans of the images of a cell co-stained for SS18, ELL3 and EP300, at the position depicted by the red line. The direction is from the top left to the bottom right. **c, d.** FRAP experiments for SS18 (**c**) and ELL3 (**d**). Left, representative images of the FRAP experiment. The white arrow refers to the punctum undergoing photobleaching. Right, quantification of FRAP data for puncta of SS18 (n = 5) and ELL3 (n = 10). The puncta were photobleached at t = 0 s and data were plot as mean $\pm$ standard error. **e-g.** GSEA-like analyses for SS18 (**e**), EP300 (**f**) and ELL3 (**g**), with all focus sites (CondSig-positive and -negative peaks of the CAP) were ranked by the $\log_2$-transformed fold change in CUT&RUN signals and annotated against the set of CondSig-positive peaks. Left, 1,6-HD treatment versus wild type, right, 1,6-HD treatment versus 2,5-HD treatment, and the pseudo count in fold change calculations was set to 0.1. **h.** UCSC genome browser view of representative CondSig-positive sites. Signals represent RPM and the related loci were shaded in purple. Source data are provided as a Source Data file.

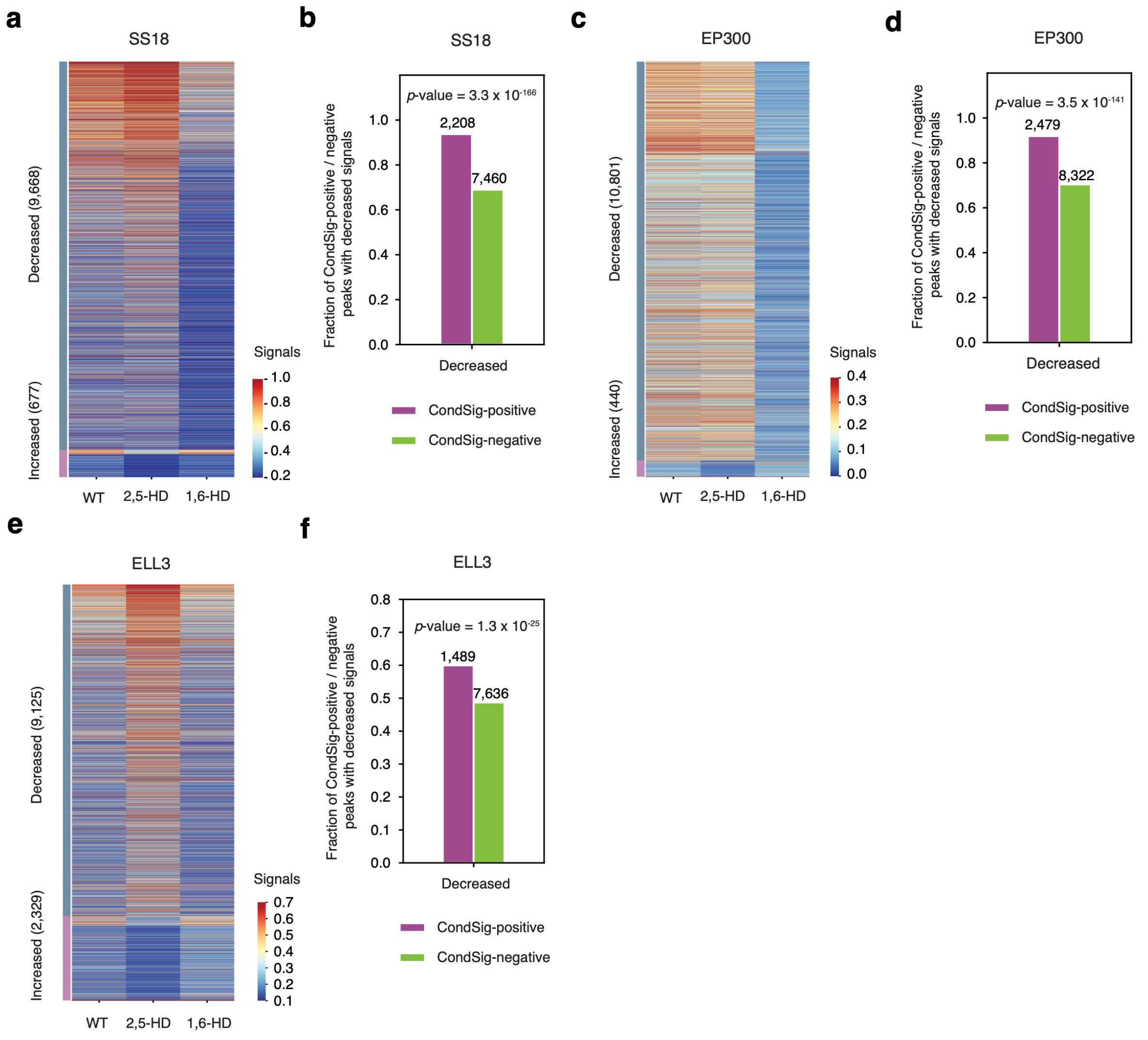

Supplementary Fig. 11. Decreases of SS18, EP300 and ELL3 after 1,6-HD treatment. **a.** Heatmap showing CUT&RUN signals for SS18 across its peaks with changed signals (including decreased and increased peaks) when comparing 1,6-HD treatment to 2,5-HD treatment. Peaks with decreased signals (log_2-transformed fold change < -0.25) were marked in dark blue and peaks with increased signals (log_2-transformed fold change > 0.25) were marked in purple. Each group of peak was ranked based on the result of a *k*-means clustering without predetermined cluster centroids, which utilized signals from both 2,5-HD treatment and 1,6-HD treatment. **b.** Bar plots showing the fractions of CondSig-postiive / negative peaks with decreased signals out of all CondSig-positive / negative peaks, the exact number of peaks was labelled on the top of bars. A Fisher's exact test was performed to test the significance of enrichment of CondSig-positive peaks in the group with decreased signals and *p*-value was explicitly labelled. **c, e.** Same as (**a**) but

for EP300 and ELL3. **d, f.** Same as (**b**) but for EP300 and ELL3. Source data are provided as a Source Data file.

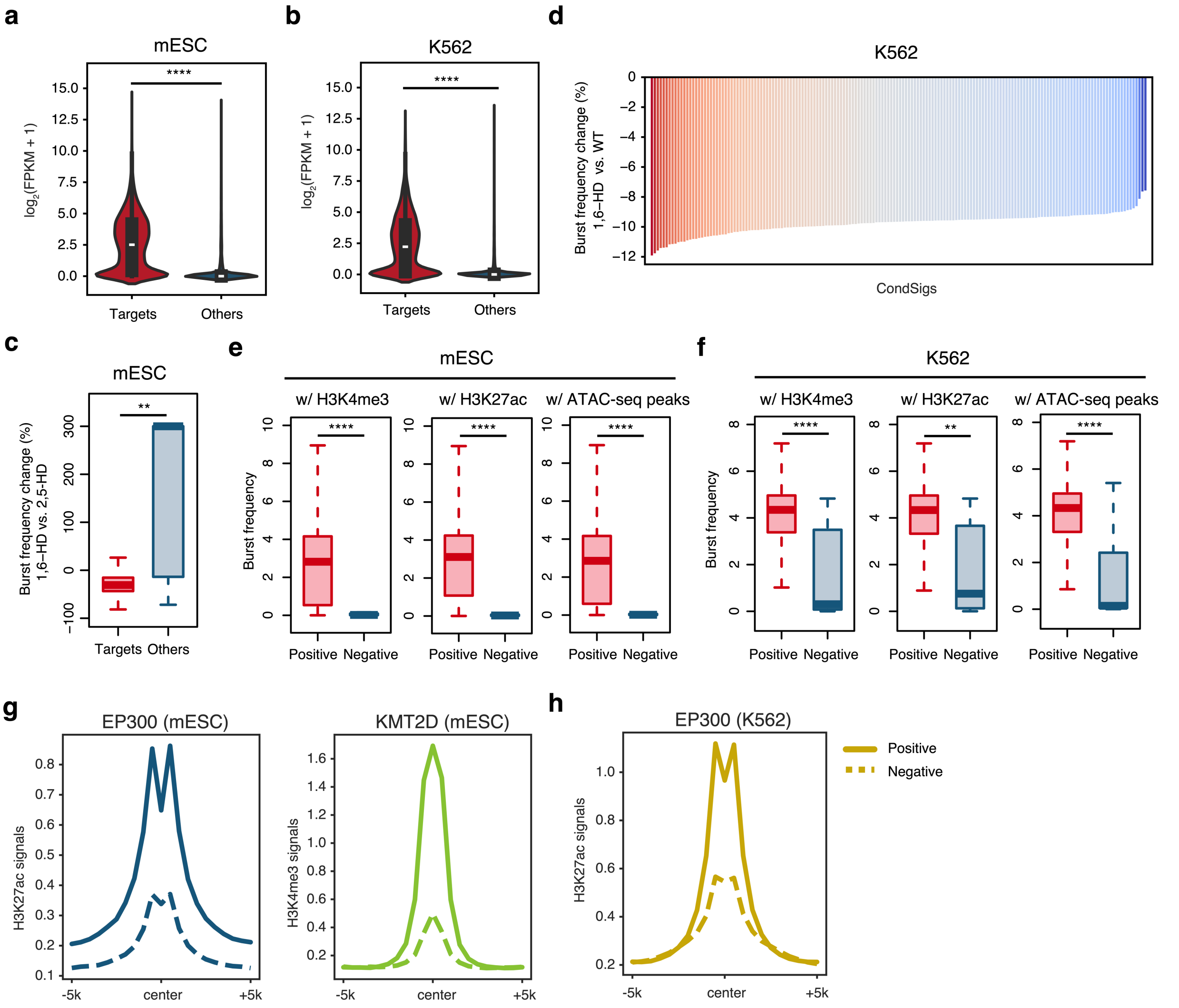

Supplementary Fig. 12. Biomolecular condensates affect transcription and histone modification regulation.

a, b. Violin plots showing expression levels of genes targeted by CondSig-positive sites and other genes in mESC (**a**) and K562 (**b**). The *Y*-axis represents $\log_2$-transformed fragments per kilobase of transcript per million mapped reads (FPKM). RNA-seq data in mESC and K562 were from ENCODE project (ENCSR000CGU, ENCSR000AEM)[9]. Significance between groups was evaluated by a one-sided Welch's *t*-test, **** represents *p*-value $< 1 \times 10^{-4}$. Sample sizes for comparison are 16,811/8,648 for target genes/other genes in mESC, and 17,837/15,612 for target genes/other genes in K562. **c**. Box plots compared the burst frequency change percentages after 1,6-HD treatment compared to 2,5-HD treatment of target genes and other genes in mESC. The centre lines mark the median, the box limits indicate the 25th and 75th percentiles, and the whiskers extend to $1.5 \times$ the interquartile range from the 25th and 75th percentiles. Significance between

groups was evaluated by a one-sided Welch's *t*-test, ** represents *p*-value < 0.01. The samples used to derive statistics are provided in Source data. **d.** The bar plots showing change percentages of burst frequency of genes targeted by each individual CondSig in K562. **e, f.** Box plots comparing burst frequencies of genes targeted by all CondSig-positive sites and all CondSig-negative sites with same histone modifications or chromatin accessibility in mESC (**e**) and K562 (**f**). Sites overlapping with H3K4me3, H3K27me3 ChIP-seq peaks or ATAC-seq peaks were analyzed. The centre lines mark the median, the box limits indicate the 25th and 75th percentiles, and the whiskers extend to 1.5 × the interquartile range from the 25th and 75th percentiles. Significance between groups was evaluated by a one-sided Welch's *t*-test, ** represents *p*-value < 0.01 and **** represents *p*-value < 1×10^{-4}. The samples used to derive statistics are provided in Source data. **g, h.** Line charts showing corresponding histone modification profiles at CondSig-positive and -negative peaks of each histone modification reader in mESC (**g**) and K562 (**h**). Source data are provided as a Source Data file.

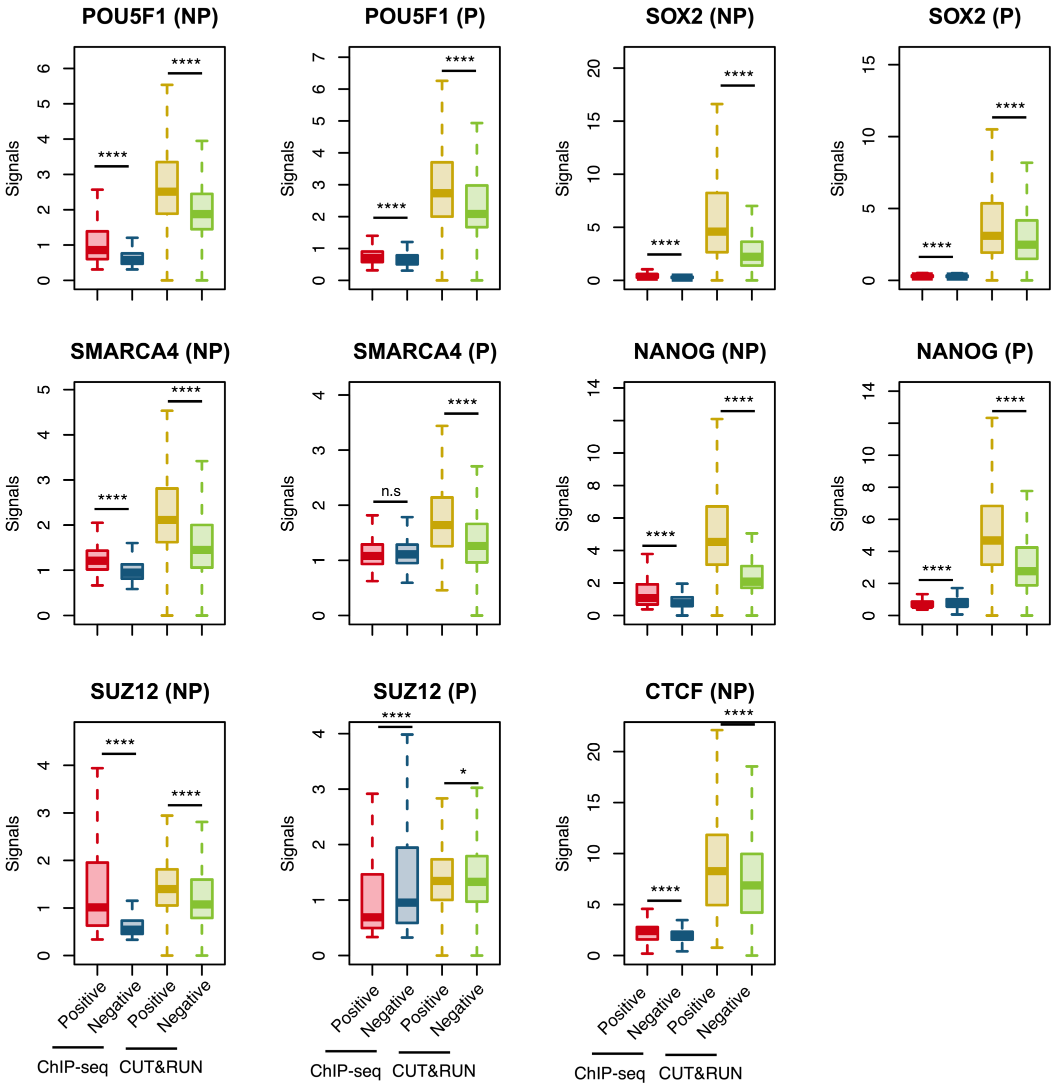

Supplementary Fig. 13. The comparison of ChIP-seq and CUT&RUN signals at CondSig-positive and -negative peaks.

Box plots showing comparisons of ChIP-seq and CUT&RUN signals at CondSig-positive and -negative peaks of several CAPs. The centre lines mark the median, the box limits indicate the 25th and 75th percentiles, and the whiskers extend to 1.5 × the interquartile range from the 25th and 75th percentiles. Significance between groups was evaluated by a two-sided Welch's *t*-test, n.s represents non-significant, * represents *p*-value < 0.05 and **** represents *p*-value < 1×10^{-4}. The samples used to derive statistics are provided in Source data. Source data are provided as a Source Data file.

Supplementary References

1 Pedregosa, F. *et al.* Scikit-learn: Machine learning in Python. *the Journal of machine Learning research* **12**, 2825-2830 (2011).

2 Hu, S. *et al.* ncHMR detector: a computational framework to systematically reveal non-classical functions of histone modification regulators. *Genome Biol* **21**, 48 (2020). https://doi.org:10.1186/s13059-020-01953-0

3 Di Stefano, B. *et al.* C/EBPalpha creates elite cells for iPSC reprogramming by upregulating Klf4 and increasing the levels of Lsd1 and Brd4. *Nat Cell Biol* **18**, 371-381 (2016). https://doi.org:10.1038/ncb3326

4 Schmidl, C., Rendeiro, A. F., Sheffield, N. C. & Bock, C. ChIPmentation: fast, robust, low-input ChIP-seq for histones and transcription factors. *Nat Methods* **12**, 963-965 (2015). https://doi.org:10.1038/nmeth.3542

5 Zheng, R. *et al.* Cistrome Data Browser: expanded datasets and new tools for gene regulatory analysis. *Nucleic Acids Res* **47**, D729-D735 (2019). https://doi.org:10.1093/nar/gky1094

6 Guo, Y. & Gifford, D. K. Modular combinatorial binding among human trans-acting factors reveals direct and indirect factor binding. *BMC Genomics* **18**, 45 (2017). https://doi.org:10.1186/s12864-016-3434-3

7 Yan, X., Guo, J., Lan, Y. & Cheng, X. in *Proceedings of the 22nd international conference on World Wide Web.* 1445-1456.

8 Tang, Z. *et al.* CTCF-Mediated Human 3D Genome Architecture Reveals Chromatin Topology for Transcription. *Cell* **163**, 1611-1627 (2015). https://doi.org:10.1016/j.cell.2015.11.024

9 Dunham, I. *et al.* An integrated encyclopedia of DNA elements in the human genome. *Nature* **489**, 57-74 (2012). https://doi.org:10.1038/nature11247
