# Supplementary material for: Decoding the genomic landscape of chromatin-associated biomolecular condensates
Source: Nat Commun. 2024 Aug 13;15:6952. doi: 10.1038/s41467-024-51426-2 (PMC11322608; doi:10.1038/s41467-024-51426-2)
Supplement: Supplementary file 3 — Description of Additional Supplementary Files [file 41467_2024_51426_MOESM3_ESM.pdf]

Description of Additional Supplementary Files

File Name: Supplementary Data 1
Description: Known chromatin-associated biomolecular condensates.

File Name: Supplementary Data 2
Description: Qualified ChIP-seq datasets for CAPs in mESC and K562.
