# Supplementary material for: Decoding the genomic landscape of chromatin-associated biomolecular condensates
Source: Nat Commun. 2024 Aug 13;15:6952. doi: 10.1038/s41467-024-51426-2 (PMC11322608; doi:10.1038/s41467-024-51426-2)
Supplement: Supplementary file 6 — Reporting Summary [file 41467_2024_51426_MOESM6_ESM.pdf]

nature portfolio

Corresponding author(s): Yong Zhang

Last updated by author(s): Jul 1, 2024

Reporting Summary

Nature Portfolio wishes to improve the reproducibility of the work that we publish. This form provides structure for consistency and transparency in reporting. For further information on Nature Portfolio policies, see our Editorial Policies and the Editorial Policy Checklist.

Statistics

For all statistical analyses, confirm that the following items are present in the figure legend, table legend, main text, or Methods section.

n/a	Confirmed	
☐	☒	The exact sample size (n) for each experimental group/condition, given as a discrete number and unit of measurement
☐	☒	A statement on whether measurements were taken from distinct samples or whether the same sample was measured repeatedly
☐	☒	The statistical test(s) used AND whether they are one- or two-sided<br>*Only common tests should be described solely by name; describe more complex techniques in the Methods section.*
☒	☐	A description of all covariates tested
☐	☒	A description of any assumptions or corrections, such as tests of normality and adjustment for multiple comparisons
☐	☒	A full description of the statistical parameters including central tendency (e.g. means) or other basic estimates (e.g. regression coefficient) AND variation (e.g. standard deviation) or associated estimates of uncertainty (e.g. confidence intervals)
☐	☒	For null hypothesis testing, the test statistic (e.g. F, t, r) with confidence intervals, effect sizes, degrees of freedom and P value noted<br>*Give P values as exact values whenever suitable.*
☒	☐	For Bayesian analysis, information on the choice of priors and Markov chain Monte Carlo settings
☒	☐	For hierarchical and complex designs, identification of the appropriate level for tests and full reporting of outcomes
☒	☐	Estimates of effect sizes (e.g. Cohen's d, Pearson's r), indicating how they were calculated

Our web collection on statistics for biologists contains articles on many of the points above.

Software and code

Policy information about availability of computer code

Data collection	Public ChIP-seq data of CAPs were collected from Cistrome Data Browser and filtrated using quality control procedures. Human and mouse proteins with reported LLPS capacity were collected from four databases, DrLLPS, LLPSDB, PhaSepDB and PhaSePro. Component proteins of MLOs in human and mouse were collected from DrLLPS and PhaSepDB. Pairwise protein-protein interactions were collected from three databases, BioGRID, MINT and IntAct, only physical associations were kept. Intrinsically disordered regions of proteins were predicted by MobiDB-lite (v1.0). RNA-binding proteins were predicted by TriPepSVM (v1.0). Motif scans were performed using FIMO (v5.0.5) against the JASPAR core 2020 vertebrates database.<br><br>Images of immunofluorescence staining were acquired using a Zeiss LSM 710 confocal microscope with 100 × oil objective and ZEN acquisition software. Fluorescence intensity of FRAP experiment was measured using Fiji software.

Data analysis	CondSigs were identified by our custom computational framework, CondSigDetector (https://github.com/TongjiZhanglab/CondSig).<br><br>CUT&RUN reads generated in this study were first processed using TrimGalore (v0.6.0) to trim adapter and low-quality reads. Trimmed reads were then aligned to the mouse genome build mm10 or human genome build hg38 using Bowtie2 (v2.3.5.1) with parameters "--no-mixed --no-discordant --no-unal". Low mapping quality reads (mapping quality < 30) and duplicates were discarded. Then biological replicates that passed quality control were pooled together. CUT&RUN peaks were called by MACS2 (v2.1.3). Signal tracks were generated using the "genomecov" command in Bedtools software (v2.28.0), and normalized to reads per million mapped reads (RPM). Single-cell RNA-seq data (10x Genomics) were processed with DrSeq2 (v2.2.0) and transcriptome-wide transcriptional burst kinetics were inferred using the model from the previous study (PMID: 30602787).

For manuscripts utilizing custom algorithms or software that are central to the research but not yet described in published literature, software must be made available to editors and reviewers. We strongly encourage code deposition in a community repository (e.g. GitHub). See the Nature Portfolio guidelines for submitting code & software for further information.

Data

Policy information about availability of data

All manuscripts must include a data availability statement. This statement should provide the following information, where applicable:

- Accession codes, unique identifiers, or web links for publicly available datasets
- A description of any restrictions on data availability
- For clinical datasets or third party data, please ensure that the statement adheres to our policy

All the CUT&RUN and scRNA-seq data generated in this study have been deposited in Genome Sequence Archive (https://ngdc.cncb.ac.cn/gsa/) under accession code CRA011710 (https://ngdc.cncb.ac.cn/gsa/browse/CRA011710) and HRA005013 (https://ngdc.cncb.ac.cn/gsa-human/browse/HRA005013). All predicted CondSigs, the associated CondSig-positive sites, and high-confidence condensate-related sites generated in this study are available at CondSigDB (https://compbio-zhanglab.org/CondSigDB/index.html). Source data are provided with this paper.

Research involving human participants, their data, or biological material

Policy information about studies with human participants or human data. See also policy information about sex, gender (identity/presentation), and sexual orientation and race, ethnicity and racism.

Reporting on sex and gender	n/a
Reporting on race, ethnicity, or other socially relevant groupings	n/a
Population characteristics	n/a
Recruitment	n/a
Ethics oversight	n/a

Note that full information on the approval of the study protocol must also be provided in the manuscript.

Field-specific reporting

Please select the one below that is the best fit for your research. If you are not sure, read the appropriate sections before making your selection.

☒ Life sciences ☐ Behavioural & social sciences ☐ Ecological, evolutionary & environmental sciences

For a reference copy of the document with all sections, see nature.com/documents/nr-reporting-summary-flat.pdf

Life sciences study design

All studies must disclose on these points even when the disclosure is negative.

Sample size	No statistical methods were used to predetermine sample size. Sample sizes were determined based on previous related studies.
Data exclusions	No data were excluded from the analyses.
Replication	CUT&RUN assay was performed 2-3 times for each sample. Single-cell RNA-seq was performed once for each condition, and the number of replicates was sufficient to perform the subsequent analysis. For each CondSig, FRAP experiments were performed 5-10 times for each component. Additionally, two independent cell-seeding, fixation, and co-IF staining experiments (three slides per experiment) were performed. All attempts at replication were successful.<br><br>The replicates of CUT&RUN assay were initiated after completing library construction from the previous sample, continuing from cell seeding to the conclusion of the full experiment. Likewise, for the co-IF experiments, replicates began with seeding the next batch of cells following the staining of the previous sample, and then completing the subsequent experiments. The replicates of FRAP assays were accomplished by extinguishing the punctum generated in distinct cells. All cells used in the experiments were from the same batch of frozen stocks.

Randomization	This study did not include complex treatment conditions, all cells were randomly assigned to each group for imaging and sequencing.
Blinding	All samples were prepared blinded.

Reporting for specific materials, systems and methods

We require information from authors about some types of materials, experimental systems and methods used in many studies. Here, indicate whether each material, system or method listed is relevant to your study. If you are not sure if a list item applies to your research, read the appropriate section before selecting a response.

Materials & experimental systems

n/a	Involved in the study
☐	☒ Antibodies
☐	☒ Eukaryotic cell lines
☒	☐ Palaeontology and archaeology
☒	☐ Animals and other organisms
☒	☐ Clinical data
☒	☐ Dual use research of concern
☒	☐ Plants

Methods

n/a	Involved in the study
☒	☐ ChIP-seq
☒	☐ Flow cytometry
☒	☐ MRI-based neuroimaging

Antibodies

Antibodies used	Rabbit DDX21 antibody (Proteintech, 10528-1-AP, lot # 00088037, 2 µl for CUT&RUN), rabbit CTR9 antibody (Bethyl Laboratories, A301-395A, polyclonal, lot # 4, 4µl for CUT&RUN and 1:250 diluted for co-IF), rabbit SUPT6H antibody (Novus Biologicals, NB100-2582, polyclonal, lot # A2, 4µl for CUT&RUN and 1:200 diluted for co-IF), rabbit SUPT5H antibody (Santa Cruz, cat # sc-133217 (D3), lot # G1217, 1:100 diluted for co-IF), rabbit SS18 antibody (Cell Signaling Technology, 21792(D6I4Z), lot # 1, 1.5 µl for CUT&RUN and 1:400 diluted for co-IF), mouse EP300 antibody (Santa Cruz, sc-48343(F-4) lot # A1323, 1.5 µl for CUT&RUN; Santa Cruz, cat # sc-32244(NM11), lot # H1921, 1:200 diluted for co-IF) and ELL3 antibody (Sigma, cat # HPA028938, polyclonal, lot# R30687, 1:200 diluted for co-IF; 0.5 µl for CUT&RUN, generously gifted by Prof. Chengqi Lin, Southeast University, China).
Validation	DDX21 https://www.ptglab.com/Products/DDX21-Antibody-10528-1-AP.htm#product-information<br>CTR9 https://www.thermofisher.cn/cn/zh/antibody/product/CTR9-Antibody-Polyclonal/A301-395A<br>SUPT6H https://www.novusbio.com/products/spt6-antibody_nb100-2582<br>SUPT5H https://www.scbt.com/zh/p/spt5-antibody-d-3<br>SS18 https://www.cellsignal.com/products/primary-antibodies/ss18-d6i4z-rabbit-mab/21792<br>EP300 (CUT&RUN) https://www.scbt.com/zh/p/p300-antibody-f-4<br>EP300 (co-IF) https://www.scbt.com/zh/p/p300-antibody-nm11<br>ELL3 (co-IF) https://www.sigmaaldrich.com/US/en/product/sigma/hpa028938<br>ELL3 (CUT&RUN) Meng, S., Liu, X., Zhu, S. et al. Young LINE-1 transposon 5' UTRs marked by elongation factor ELL3 function as enhancers to regulate naïve pluripotency in embryonic stem cells. Nat Cell Biol 25, 1319–1331 (2023).https://doi.org/10.1038/s41556-023-01211-y

Eukaryotic cell lines

Policy information about cell lines and Sex and Gender in Research

Cell line source(s)	Mouse embryonic stem cells (ATCC (SCRC-1002), K562 (National Collection of Authenticated Cell Cultures ,TCHu191). Sex was not considered in this study.
Authentication	Mouse embryonic stem cells were authenticated by ATCC, and K562 cells were authenticated by National Collection of Authenticated Cell Cultures.
Mycoplasma contamination	All cell lines used tested negative for mycoplasma contamination using the MycoAlert Detection Kit (Lonza, LT07-318).
Commonly misidentified lines (See ICLAC register)	No cell lines from the ICLAC register were used.

Plants

Seed stocks	n/a
Novel plant genotypes	n/a
Authentication	n/a
